# Supplementary material for: Spatiotemporal Distribution and Evolution of Digestive Tract Cancer Cases in Lujiang County, China since 2012
Source: Int J Environ Res Public Health. 2022 Jun 17;19(12):7451. doi: 10.3390/ijerph19127451 (PMC9223376; doi:10.3390/ijerph19127451)
Supplement: Supplementary file 1 [file ijerph-19-07451-s001.zip › ijerph-1700394-supplementary.pdf]

Supplementary materials

Table S1. The results of Kulldorff's space-time scan statistical analysis.

Summary of data	Most likely cluster	Secondary cluster
Locations township	Jinniu, Guohe, Wanshan, and Tangchi Town	Luohe, Nihe, and LeqiaoTown
Coordinates	31.399702N, 117.203274E	31.015947N, 117.278636E
Radius	8.23km	16.33km
Time frame	2015/1/1 to 2017/12/31	2013/1/1 to 2014/12/31
Population	184380	226860
Number of cases	1472	1089
Expected cases	1164.62	949.95
Annual cases/100000	265.0	240.3
Observed/expected	1.26	1.15
Relative risk	1.29	1.16
Log likehood ratio	40.824557	10.401037
P-value	0.00000000000000067	0.0021

Table S2. Amount of pesticide and fertilizer used in Lujiang County.

Town	Fertilizer (t)	Ammonia Fertilizer(t)	Phosphate Fertilizer (t)	Potash fertilizer (t)	Compound fertilizer (t)	Pesticide (t)
Baihu	11072	2387	982	2880	4822	134
Baishan	2817	608	250	733	1227	34
Fanshan	1666	359	148	433	726	20
Guohe	4058	875	360	1055	1767	49
Jinniu	2532	546	225	659	1103	31
Ketan	3055	659	271	795	1331	37
Leqiao	3353	723	297	872	1460	40
Longqiao	1931	416	171	502	841	23
Lucheng	3201	690	284	833	1394	39
Luohe	2653	572	235	690	1156	32
Nihe	4753	1025	422	1236	2070	57
Shengqiao	3314	715	294	862	1443	40
Shitou	2689	580	239	699	1171	32
Tangchi	2291	494	203	596	998	28
Tongda	4591	990	407	1194	2000	55
Wanshan	2605	562	231	678	1135	31
Yefushantown	3526	760	313	917	1536	43

Table S3. Ellipse parameters of standard deviation of digestive tract cancer incidence in Lujiang County from 2012 to 2017.

Year	Xcoord	Ycoord	XStdDist/km	YStdDist/km	Rotation	Shape area/km^2
2012	117.283084	31.281476	15.41227	22.51078	174.275707	1089.88
2013	117.281104	31.279980	15.59267	22.61495	170.832485	1107.74
2014	117.286161	31.275593	15.46771	23.19220	170.565837	1126.91
2015	117.281941	31.282448	15.44344	22.69382	171.114564	1100.97
2016	117.280623	31.285788	15.34864	22.43918	169.846305	1081.93
2017	117.277296	31.290066	15.35908	22.47335	170.24876	1084.32

Table S4. GM(1,1) model test results.

Town	Residual	Relative error	Level ratio deviation
Baihu	0.0997	0.0227	0.0466
Baishan	0.0000	0.0266	0.0550
Fanshan	0.0380	0.0664	-0.0132
Guohe	0.0163	0.0410	0.0742
Jinniu	0.0267	0.0860	-0.0090
Ketan	0.0185	0.0310	0.0190
Leqiao	0.0568	0.0532	0.0402
Longqiao	0.0002	0.0923	-0.0002
Lucheng	0.0390	0.0114	-0.0244
Luohe	0.0760	0.0188	0.0306
Nihe	0.0188	0.0536	0.0356
Shengqiao	0.0407	0.0461	0.0074
Shitou	0.0012	0.0644	-0.0032
Tangchi	0.1238	0.0693	0.0264
Tongda	0.0582	0.1000	-0.0820
Wanshan	0.0002	0.0696	-0.0274
Yefushan	0.0165	0.0564	-0.0210
Total	0.0371	0.0535	0.0091

Note: The GM(1,1) model is mainly used to test residuals, including relative error and step ratio deviation. In this study, the relative error and the level ratio deviation are both ≤0.1, and the accuracy grades are both level I, indicating that the prediction results of the GM(1,1) model are highly reliable.

Table S5. Ellipse parameters of standard deviation of digestive tract cancer incidence in Lujiang County from 2018 to 2025.

Year	Xcoord	Ycoord	XStdDist	YStdDist	Rotation	Shape_area/km^2
2018	117.277054	31.286751	15.25476	22.32633	168.263567	1069.91
2019	117.275474	31.292448	15.18283	22.18749	167.773938	1058.24
2020	117.273816	31.295195	15.10749	22.03643	167.299768	1045.82
2021	117.272079	31.297995	14.94629	21.69436	166.403244	1018.60
2022	117.270264	31.300848	14.94629	21.69436	166.403244	1018.60
2023	117.268372	31.303754	14.86022	21.50158	165.984706	1003.73
2024	117.266403	31.306713	14.77043	21.29300	165.588877	987.99
2025	117.26436	31.309725	14.67678	21.06763	165.218326	971.34

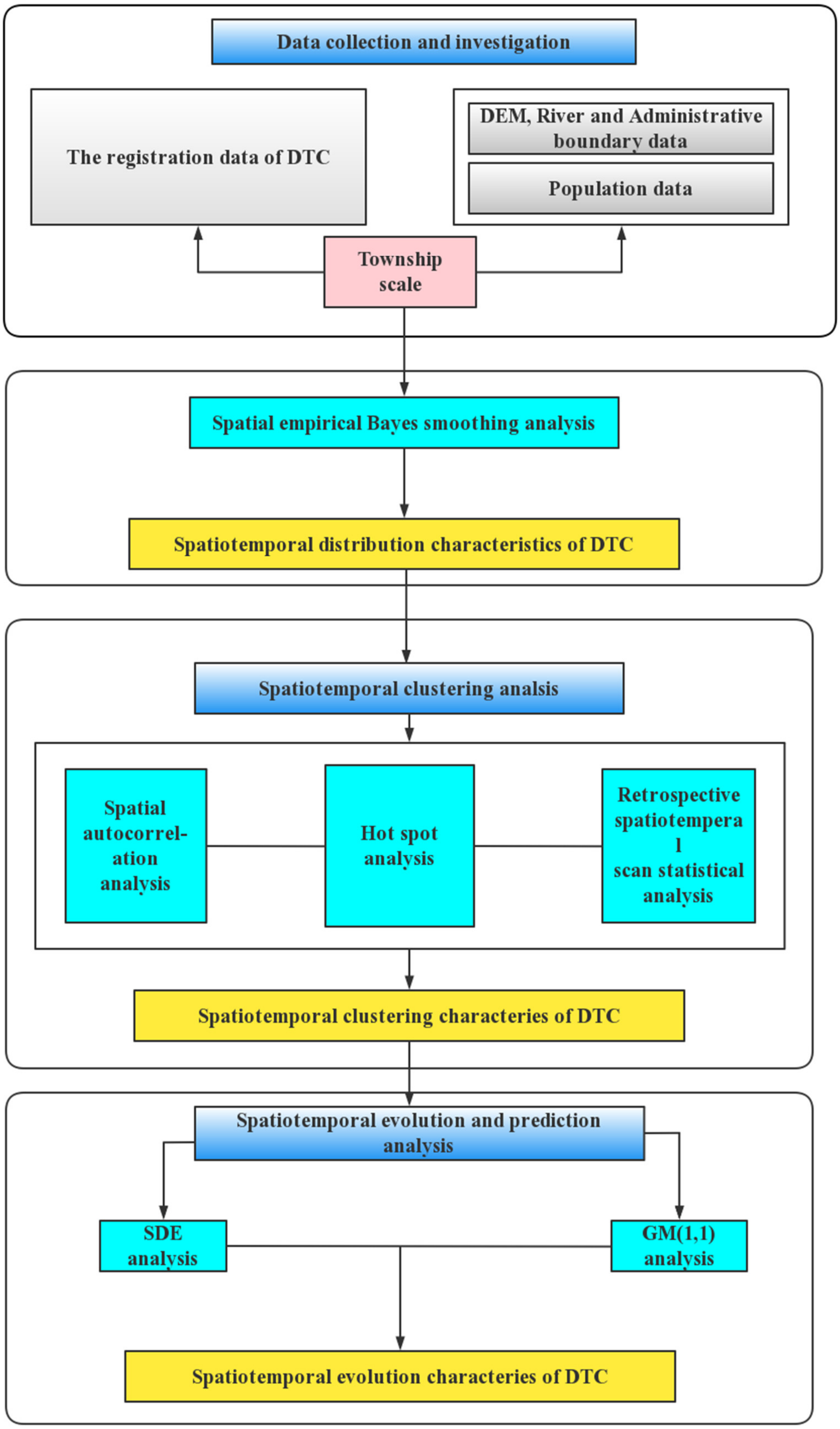

Figure S1. Research framework.

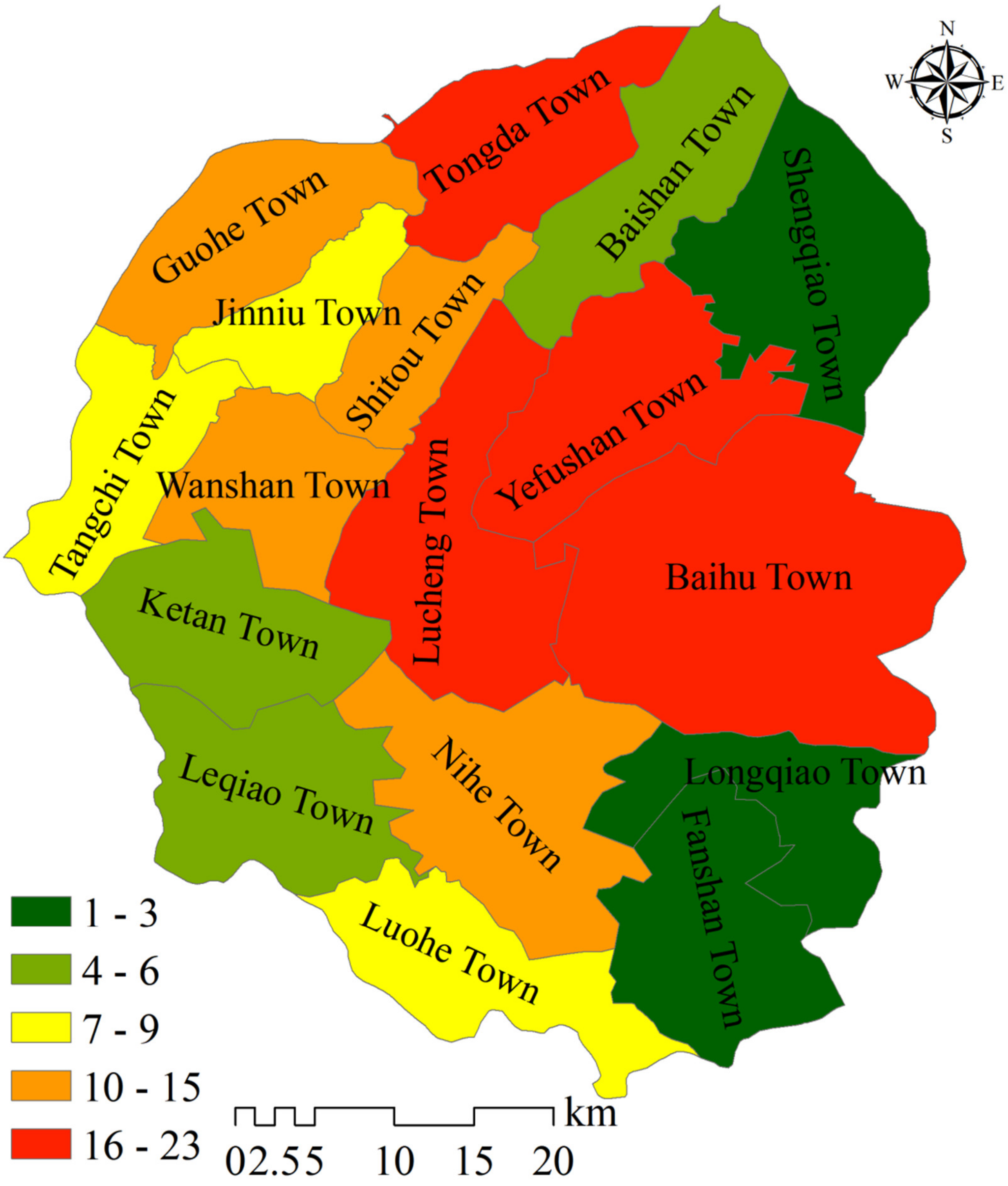

Figure S2. Number of industrial enterprises above designated size (annual operating income> 20 million).
